# Quantitative Imaging Analysis of FDG PET/CT Imaging for Detection of Central Neurolymphomatosis in a Case of Recurrent Diffuse B-Cell Lymphoma.

**DOI:** 10.7759/cureus.379

**Published:** 2015-11-13

**Authors:** Faiq Shaikh, Derek Savells, Omer Awan, Faisal Inayat, Ammar Chaudhry, Nivedita Jerath, Michael M Graham

**Affiliations:** 1 Imaging Informatics, University of Pittsburgh Medical Center; 2 Molecular Imaging Physician, S&L Readings, LLC.; 3 Department of Radiology, University of Iowa Hospitals and Clinics; 4 Department of Radiology, Dartmouth Hitchcock Medical Center; 5 Department of Medicine, New York-Presbyterian Hospital, Weill Cornell Medical College, New York, N.Y., USA; 6 Neuroradiology, Johns Hopkins University School of Medicine; 7 Department of Neurosurgery, University of Iowa Hospitals and Clinics

**Keywords:** neurolymphomatosis, fdg pet/ct, pneuro, diffuse large b-cell lymphoma (dlbcl), quantitative, analysis

## Abstract

Neurolymphomatosis (NL) is a rare disease characterized by malignant lymphocytes infiltrating various structures of the nervous system. It typically manifests as a neuropathy involving the peripheral nerves, nerve roots, plexuses, or cranial nerves. It often presents as a complication of lymphoma, but it can be the presenting feature of recurrent lymphoma. It is essential to identify and initiate treatment early with chemotherapy and/or radiation therapy in all cases of nodal or visceral (including neural) involvement with lymphoma. There are various diagnostic tests that can be used for its detection, such as cerebrospinal spinal fluid (CSF) cytology, electromyography (EMG), magnetic resonance imaging (MRI), and positron-emission tomography/computed tomography (PET/CT). FDG-PET/CT is the standard of care in lymphoma staging, restaging, and therapy response assessment, but has an inherent limitation in the detection of disease involvement in the central nervous system. While that is mostly true for visual assessment, there are quantitative methods to measure variation in the metabolic activity in the brain, which in turn helps detect the occurrence of neurolymphomatosis.

## Introduction

We present a case where neural involvement with diffuse large B-cell lymphoma (DLBCL) was suspected clinically in a patient otherwise thought to be in remission. Based on the concern raised by MRI findings, an FDG PET/CT scan was performed, which revealed abnormal metabolic activity in the brain on quantitative image analysis using the PMOD-PNeuro^R^ tool (PMOD Technologies, Zurich, Switzerland).

## Case presentation

Signed informed patient consent was obtained for this patient's care.

**Figure 1:**
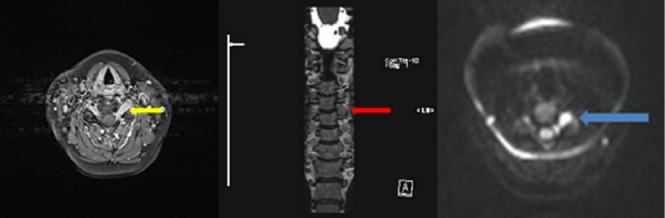
Initial MRI on presentation *Left to right* - *1st image: *Axial T1-weighted fat-sat post-contrast image shows asymmetric enlargement and uniform enhancement of the left C4-5 nerve root (yellow arrow).* 2nd image: *Corresponding coronal T2-weighted image showing the lesional enhancement (red arrow). *3rd image - *The nerve root shows intense diffusion restriction of diffusion weighted imaging (blue arrow).

We present a case of a 48-year-old female with an existing diagnosis of Stage IV diffuse large B-cell lymphoma (DLBCL), in remission as per a surveillance 18F-fluorodeoxyglucose positron emission tomography/computed tomography (FDG-PET/CT) scan, who presented with left arm weakness after a fall. The initial magnetic resonance imaging (MRI) revealed enhancement in two upper cervical roots (Figure 1).

**Figure 2:**
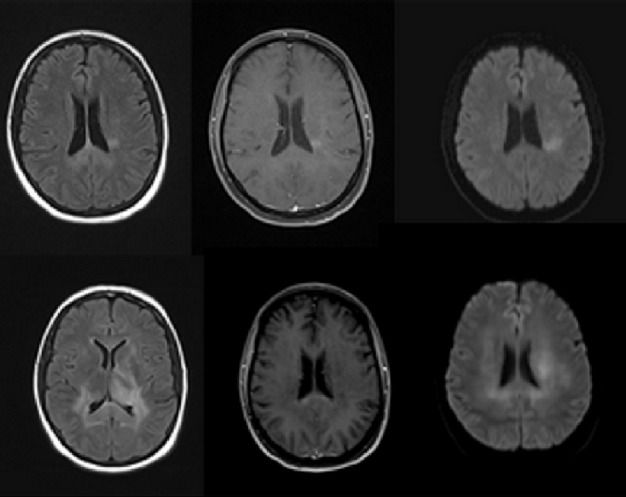
Comparison of initial and follow-up MR images Images from the initial *(top row) *and subsequent MRI *(bottom row) *demonstrating an additional site of lymphomatous involvement. Abnormal flair intensity *(1st column)* and abnormal patchy enhancement is seen on post-contrast images in the periventricular white matter and corpus callosum *(second column) *with corresponding DWI restriction *(third column).*

Electromyographic (EMG) studies demonstrated good sensory potentials, but fibrillations were noted in the muscles innervated by C5-6. Thus, the working diagnosis was cervical plexitis/radiculitis, and treatment with Neurobion (vitamin supplements) was initiated. However, the patient’s symptoms continued to worsen in the ensuing month with the new development of right shoulder pain. A repeat cervical spine MRI was performed, which revealed an enhancing mass along the left C5 nerve root suggestive of a neurogenic tumor. A lumbar puncture was then performed and cytology of the cerebrospinal fluid (CSF) revealed lymphocytosis without evidence of tumor cells. Neurosurgery was consulted, and a laminectomy was performed, along with the excision of the cervical root mass, the pathology of which revealed DLBCL. This prompted an MRI of the brain, and it demonstrated periventricular and callosal hyperintense foci with faint enhancement and diffusion-weighted hyperintensity (Figure 2), which were felt to be concerning for lymphoma. A repeated LP showed the presence of lymphoma cells. The bone marrow biopsy was negative for malignancy.

**Figure 3:**
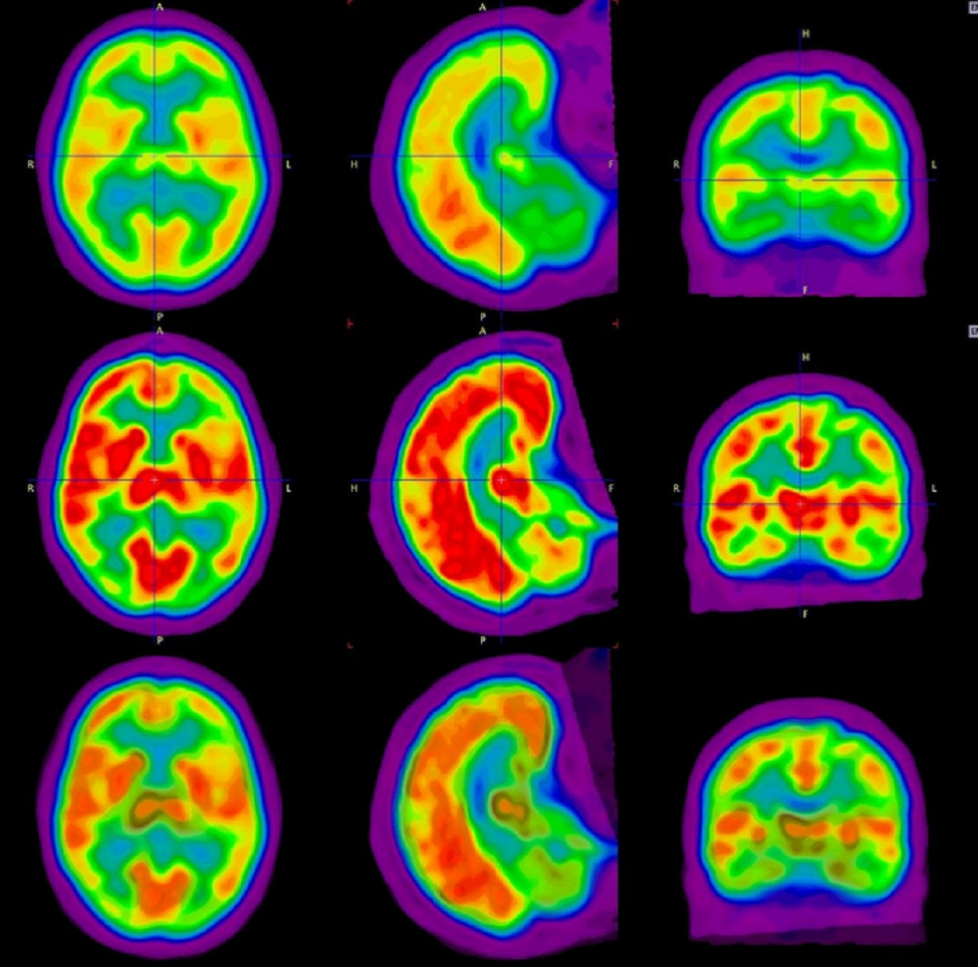
Comparison of initial and follow-up PET/CT images using PMOD/PNeuro tool The initial PET/CT demonstrates no areas of abnormal uptake to suggest brain involvement *(top row).* The subsequent PET/CT demonstrates patchy areas of intensely increased uptake *(middle row)*. Subtraction images show difference increase in uptake suggestive of lymphomatous infiltration *(third row). *The analysis performed suggested a heterogeneous pattern of uptake in the cortical gray matter and callosal white matter​, and many areas corresponded to abnormalities detected by MRI.

Based on this clinical picture and the suggestive findings on MRI, an FDG-PET/CT was performed. However, there were no visually apparent hypermetabolic lesions on initial assessment. However, acknowledging the fact that FDG physiologically accumulates in the brain tissue and an otherwise hypermetabolic lesion could be obscured, brain metabolic activity was further assessed using the PMOD/PNeuroR software application (Figure 3). PMOD/PNeuroR software is a quantitative image analysis tool that performs quantification of variations in the FDG activity (on PET/CT) in different regions of the brain (based on MRI parcellation), which are expressed on a color spectrum as standard deviations from the normal PET database. The application of this tool revealed an abnormally increased heterogeneous pattern of tracer uptake in various regions of the brain (Table 1), which was suggestive of disease involvement. Areas, such as the thalami, demonstrated correlative abnormal findings on MRI and the quantitative PET assessment. The patient was started on hyper-CVAD (cyclophosphamide, vincristine, Adriamycin, and dexamethasone) and intrathecal methotrexate with an ara-C regimen.

**Table 1:** Data from the quantitative image analysis of differential metabolic activity using PNeuro The select areas of the brain demonstrating greatest positive differential uptake included hippocampal and parahippocampal regions, as well as thalamic and basal nuclei. (SUVbw = Standardized Uptake Value - using body weight).

Brain Region (Select)	Difference SUVbw (g/m)	% Difference
Hippocampus, right	0.15724489	17.9
Hippocampus, left	0.18969916	22.0
Amygdala, right	0.16699445	20.6
Amygdala, left	0.19704809	24.1
Parahippocampus, right	0.16610518	20.7
Parahippocampus, left	0.19538376	24.5
Nucleus accumbens, right	0.30946808	28.6
Nucleus accumbens, left	0.30160365	26.2
Caudate nucleus, right	0.15631517	17.4
Caudate nucleus, left	0.12730549	14.3
Thalamus, right	0.2861532	36.4
Thalamus, left	0.18329167	19.0
Substantia nigra, right	0.21899194	25.8
Substantia nigra, left	0.21431143	23.7

## Discussion

Neurolymphomatosis is a rare manifestation of hematologic malignancies characterized by an infiltration of the cranial nerves, peripheral nerves, nerve roots, or nerve plexuses by malignant lymphocytes [1]. It causes progressive and painful axonal polyneuropathy and may occur as the initial manifestation, complication, or the relapsing feature of non-Hodgkins lymphoma (NHL). Leptomeningeal involvement is seen in at least half of the reported cases [2]. Approximately 80% of the reported neurolymphomatosis (NL) cases originate from B-cells, most of which represent aggressive disease [3]. Involvement of the nervous system by NHL is seen in approximately 10-25% of the cases [3-5].

Given the heterogeneous pattern of its involvement in the central nervous system and the limited methods available to detect brain tissue involvement, NL may be exceptionally difficult to diagnose. FDG PET/CT is routinely performed as the modality of choice for staging, restaging, therapy response, and remission assessment of NHL [6]. It is highly useful in the detection of clonal mutation that renders the indolent variants of disease to be more aggressive, warranting a change in management [7]. However, FDG PET/CT is limited in the evaluation of hypermetabolic disease involvement in the central nervous system, owing to the brain's avid glucose dependence, resulting in intensely increased uptake of FDG [8]. This results in a high tumor-to-background ratio within the brain and obscures the detection of hypermetabolic foci of disease involvement therein, especially if it is patchy in distribution. MRI, on the other hand, can suggest a lymphomatous involvement of the CNS fairly well, as suggested by hypointense signal intensity on T1-weighted and iso- to hyperintense signal on T2-weighted images, and intense homogeneous gadolinium enhancement and restricted diffusion [9]. 

In our patient, the MRI demonstrated periventricular and callosal hyperintense foci with faint enhancement and diffusion restriction, which were concerning for foci of neural recurrence of DLBCL. While the PET/CT was performed to evaluate the extent of recurrence elsewhere in the body, special attention was paid to the PET imaging features of the nervous system based on the concerns raised by the preceding MRI features. Conventionally, visual assessment of the brain (on axial slices) and the spinal cord (on sagittal slices) on PET/CT provides an overview of the general biodistribution of FDG in the central nervous system [10]. Subtle changes are considered nonspecific and only major differences in tracer distribution are generally reported, such as those secondary to cerebrovascular stroke with penumbra or encephalomalacia [11]. This is primary due to the fact that the neurons utilize glucose for their energy needs independent of insulin requirement, which renders them FDG-avid irrespective of the postprandial/fasting state [12]. While the peripheral nervous system follows the same physiologic principles, the signal count is too low for physiologic or pathologic activity to be demonstrated by cranial or peripheral nerves on PET imaging; however, a number of cases with intensely FDG-avid peripheral neurolymphomatosis have been reported [13].

In this case, as prompted by the abnormal MRI findings suggestive of lymphomatous infiltration, the decision was made to perform a quantitative assessment of the metabolic activity in the brain using a software application called PMOD (PNEURO). Quantitative assessment of the metabolic activity as a measure of malignant infiltration using various software applications is commonplace in cancer imaging [14]. PMOD/PNEURO creates an activity map of the patient’s brain through MRI parcellation for anatomic correlation, and the patterns of activity are depicted as a number of standard deviations from the mean activity in a particular region based on a normal PET database [15]. The analysis performed in this case suggested a heterogeneous pattern of uptake in the cortical gray matter and callosal white matter and was highly suggestive of disease involvement. While there were a number of brain regions with concurrent abnormal findings on both MRI and PET scans, some variations were observed between the two modalities, which may be attributable to the difference in their sensitivities and detection methods and to the variations in the metabolic and anatomic manifestations of the disease. 

Histopathologic correlation, while being the gold standard for diagnosis, is difficult to obtain with CNS lesions [16]; this case demonstrates the use of quantitative functional image analysis as an adjunct to anatomic imaging in order to provide reasonable confidence in the diagnosis of NL, especially when a biopsy is precluded. Through this example. we highlight the methodological pitfall of PET-CT when it comes to reader’s reliance on visual assessment of certain regions, such as the central nervous system, which is perhaps more expedient but can potentially overlook subtle variation in FDG uptake patterns and provides the example of the use of visual assessment as a viable and superior alternative. 

## Conclusions

Utilizing analytical tools for quantitative assessment of PET imaging features, such as PMOD/PNEURO^R^, should be considered for accurate detection when infiltrative disease involvement is suspected in organs that show high background FDG activity as is the case with the central nervous system. Innovative tools such as these are becoming increasingly commonplace and many of them are getting approval for clinical use, their judicious use can improve the diagnostic accuracy of disease processes.

## Appendices

PNeuro and PMOD are registered trademarks of PMOD Technologies, Zurich, Switzerland.
